# Viewing Patterns and Perspectival Paintings: An Eye-Tracking Study on the Effect of the Vanishing Point

**DOI:** 10.16910/jemr.13.2.15

**Published:** 2021-08-27

**Authors:** Arthur Crucq

**Affiliations:** Leiden University Centre for the Arts in Society, Leiden, The Netherlands

**Keywords:** Eye-tracking, linear perspective, vanishing point, viewing patterns, painting, geometry, visual features, attention, fixation, heatmap, Piero della Francesca, Hans Vredeman de Vries

## Abstract

Linear perspective has long been used to create the illusion of three-dimensional space on the picture plane. One of its central axioms comes from Euclidean geometry and holds that all parallel lines converge in a single vanishing point. Although linear perspective provided the painter with a means to organize the painting, the question is whether the gaze of the beholder is also affected by the underlying structure of linear perspective: for instance, in such a way that the orthogonals leading to the vanishing point also automatically guides the beholder’s gaze. This was researched during a pilot study by means of an eye-tracking experiment at the Lab for Cognitive Research in Art History (CReA) of the University of Vienna. It appears that in some compositions the vanishing point attracts the view of the participant. This effect is more significant when the vanishing point coincides with the central vertical axis of the painting, but is even stronger when the vanishing point also coincides with a major visual feature such as an object or figure. The latter calls into question what exactly attracts the gaze of the viewer, i.e., what comes first: the geometrical construct of the vanishing point or the visual feature?

## Introduction

In Western art, from the fifteenth century onwards linear perspective became conceived as the proper method to depict three-dimensional space on a flat surface. Besides being used in painting, linear perspective was also used to create images for books, such as scientific illustrations. Being invented shortly before the invention of book print, linear perspective spread rapidly throughout Europe (13). In 1435, Florentine architect Leon Battista Alberti wrote the treatise *De Pictura* in which he outlined a practical geometry for painters relying on theorems that were drawn from both geometry and optics (21). One of the central axioms on which linear perspective is based holds that all rays of light reflected from bodies, objects and surfaces converge in a single point, namely the eye of the viewer. These rays can be conceptualized as a visual pyramid. If one were to place a plane between the object and the viewer, one could catch all the rays from the object and draw the exact outline of the object as if seen from the perspective of the viewer. Conversely, the orthogonals from the bodies and objects on the picture plane will commence in a vanishing point that is at the exact opposite of the viewer’s point of perspective. The receding parallels of objects and surfaces in the painting should thus produce a credible illusion of depth on the two-dimensional surface (1; 4; 5; 7; 12; 19). As such, the vanishing point can be considered as a central point of focus in a perspectival painting. It can therefore be assumed that the vanishing point might also affect the way the beholder looks at the painting and navigates the painting (7). Psychologist Michael Kubovy (16) argues that, besides being a means to structure the representation of three-dimensional space on a two-dimensional surface, perspective is also a way to draw the viewer’s attention to the main content of the painting. In many paintings by such artists as Piero della Francesca, Leonardo da Vinci and Raphael it is indeed striking to note that important figures or actions coincide with the vanishing point (16; 23). The orthogonals, exemplified in the architectural details of the painting or in the tiles of the floors, would in this way lead the viewer’s gaze almost automatically to the vanishing point and thus to the main content (16; 28). For instance, in Raphael’s famous *School of Athens,* the vanishing point is just in between the figures of Plato and Aristotle and on Leonardo da Vinci’s *The Last Supper* the vanishing point coincides with the right eye of the head of Christ (García-Salgado, 2008). Leo Steinberg (22) argues that Christ not only tends to draw the viewer into the painting but that the pictorial space itself also appears to expand exactly from Christ. According to Rudolf Arnheim (1), even in modern non-perspectival paintings, such as Henry Moore’s *Tube Shelter Perspective*, the supposed tunnelling effect of converging lines would tend to draw the viewer into the painting towards a point of compression.

However, from the perspective of the beholder, the question of whether the orthogonals of perspectival paintings really draw the view towards the vanishing point and thus to the main content presents a methodological problem on how to find out what comes first. Is the eye indeed guided towards the vanishing point as a marker for important content, or is it, in the case of Leonardo da Vinci and Raphael, simply the head of Christ or the figures of Plato and Aristotle that draw attention? In other words, is it the compositional structure (in the case of linear perspective determined by the organisation of the illusion of three-dimensional space) that guides the viewer towards bodies and objects depicted, and through that to the content, or is it the form of those bodies and objects that makes the viewer successively aware of the compositional structure (26)? How to disentangle the two?

**Figure 1.**
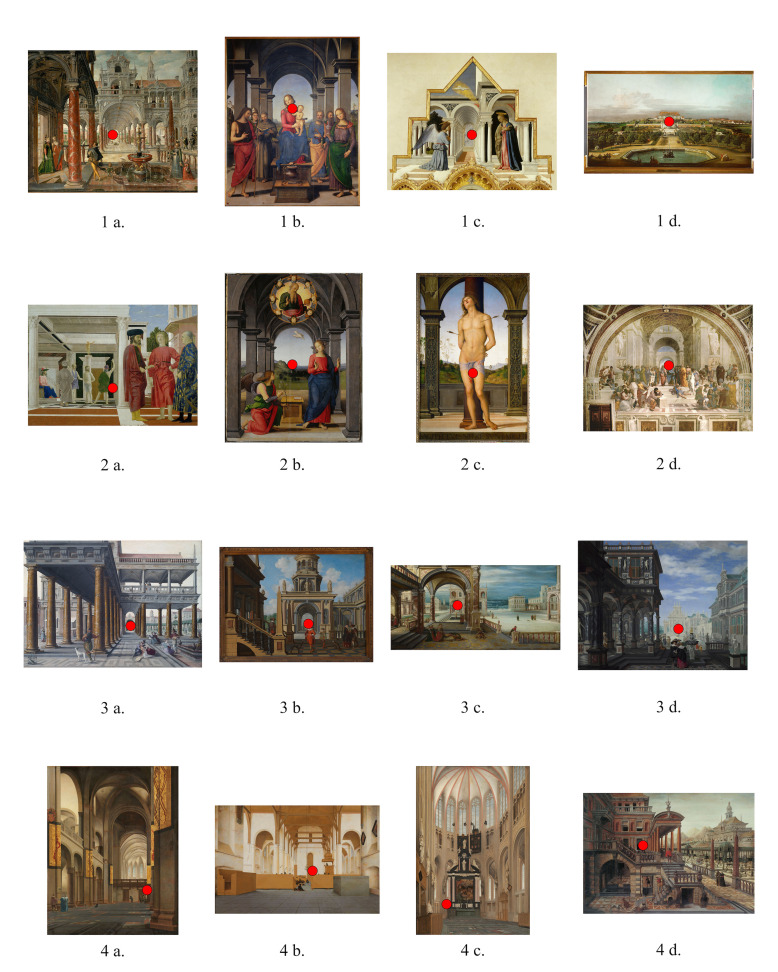
selection of paintings task I (vanishing point area indicated by experimenter)

The effect of linear perspective on viewing patterns of participants looking at perspectival paintings has been tested by means of eye tracking by Kapoula, Bucci, Yang and Bacci (11). Their experiment included the *Annunciation* from the Saint Anthony Polytech by Piero della Francesca. The authors claimed when participants were confronted with a reproduction of the image, they would in the early stages of viewing tend to fixate more on the architectural details of the painting as opposed to the important figures of Mary and the archangel Gabriel. In other words, participants would focus more on the spatial construction of the painting rather than on the narrative structure. In the case of this particular painting, the vanishing point as part of the spatial structure is located within the background of the central part of the painting where one can view an arcade with a blind marble-like wall flanked by two colonnades (see Figure 1: 1c; also Figure 5). The study by Kapoula, Bucci, Yang and Bacci (11) could thus indicate that, regardless of whether important content is placed at or near the central vanishing point, it is possible to distinguish between attention for spatial as opposed to narrative elements, at least in the first instances of viewing. Another more recent study claims that the vanishing point captures the attention of viewers and that this is not affected by bodies and objects, but in this study the researchers worked with self-made photographs and schematic grey and white depth cues which are not comparable to actual paintings from European art history (24).

In the study by Kapoula, Bucci, Yang and Bacci (11), the effect of only two paintings, both by Piero della Francesca, was investigated on the basis of a limited number of five participants. This calls for a more refined perspective on the extent to which a spatial structural component such as the vanishing point affects the viewing behaviour of the beholder and under what conditions this occurs.

The eye-tracking study described in this article aims to examine the attention paid by the viewer to the vanishing point. Compared to Kapoula, Bucci, Yang and Bacci (11) this study is based on a larger number of paintings as stimuli shown to a larger number of participants. This study was conducted during a three-month fellowship at the Lab for Cognitive Research in Art History (CReA) at the University of Vienna in the winter semester of 2017-2018. In view of the limited possibilities within the scope of the fellowship, this study was set up as a pilot study. Its objective was to collect more eye-tracking data with which to refine the research problem of how to understand the extent to which viewers are attracted to the vanishing point and how this could relate to attention given to the content of paintings. The main concerns will be addressed in the discussion session and should form the point of departure for further research that should also include a more elaborate statistical analysis of the eye-tracking data which within this pilot was not feasible.

The hypothesis underlying the pilot study holds that the vanishing point affects the viewing patterns of the beholder such that the beholder is attracted to the vanishing point and that this effect is stronger when the vanishing point coincides with the central vertical axis of the painting and with a visual feature such as a figure, an object or an architectural detail (for example, an arch, arcade or a window). Apart from the art historical examples discussed above that show that important content is often placed at the vanishing point, with regard to the central vertical axis, the hypothesis is based on, for instance, renaissance artist Piero della Francesca’s assumption that the central placement of the vanishing point would ensure the best viewing condition for the perspectival painting (4), and on the observation that many compositions in art indicate a preference for symmetrical compositions (18). The hypothesis also relies on studies that confirm a tendency of human participants to focus on the centre when looking at scenes (2).

## Methods

The effect of the vanishing point on viewing patterns was measured in this pilot study by showing the participants thirty-two reproductions of paintings from the Western tradition of art made between 1459-1761 (see Appendix). High-quality reproductions were shown in a randomized order on an Apple monitor with a resolution of 2560 x 1600. The eye movements of the participants were traced using the binocular remote eye-tracker SMI IViewX RED 120. The collected data was processed using the Eyetrace software (14). The raw data recorded by the eye-tracker was preprocessed with EyetraceButler, a plug-in for Eyetrace which converts the data into a format common for most eye-tracking devices and which can be further analyzed with Eyetrace and also shared with common statistics software. Eyetrace standard algorithm for separating fixations from saccades was used with a minimum duration time of 80 ms and a maximum radius of 100 pixels and a maximum of two measuring points as outliers to identify fixations. Eyetrace was also used to visualize fixations. Furthermore, Eyetrace was used to calculate and visualize data generated Areas of Interest (8).

### Participants

Eighteen participants were recruited at the Department of Art History of the University of Vienna. Fifteen of them were female and three were male. Their average age was 23,4 years and their mean age was 23 years during the time of the recruitment. The participants received fifteen euros for participating in the study. They all studied at the department of Art History at the time of the experiment and were of European origin. As they were all recruited within the Department of Art History, it was expected that most of them would be familiar with the method of linear perspective and that this knowledge would unconsciously affect how they looked at paintings. Moreover, it has been suggested that people trained in looking at compositional structure are better at distinguishing between aspects of form and content. A balanced perspectival construction could thus support the attention paid to the content of the painting by those familiar with linear perspective (18). A comparison with participants who are not familiar with linear perspective was not feasible within the scope of this pilot and should therefore be a key feature of follow-up research.

### Materials & design

The main variable measured was the vanishing point. The vanishing point is a geometrically determined point on the horizon of a painting (for instance a landscape or cityscape, a street view, an architectural setting), where all the orthogonal lines of the painting commence (21). Although not always immediately visible, this point can be located by following these different orthogonal lines back to the point where they commence. To study the attention paid to the vanishing point, thirty-two high-quality reproductions of paintings were selected from a period between 1435, when Alberti’s treatise on linear perspective was published in Florence, and approximately 1800. The vanishing point area in all these paintings was located by the experimenter in advance (see Figure 1 & 3). This was not indicated on the digital reproductions shown to the participants during the experiment.

For the experiment, all the reproductions of paintings selected were transferred, using Photoshop, to a slide with a light grey background. The dimensions of each projected painting were adjusted to the size of the slide (2560 x 1600). Depending on the proportions of the painting’s dimensions, either the width or the height of the reproduction was adjusted to fit the slide. As a result, differences in the size of the original paintings were disregarded.

The selection was based on four predefined possible positions of the vanishing point within the composition of a painting: 1) the central vanishing point coincides with both the central vertical axis of the painting and a visual feature such as (part of) an object, a figure or architectural detail (Figure 1: 1a – 1d); 2) the vanishing point coincides with the central vertical axis of the painting but not with a visual feature (Figure 1: 2a – 2d); 3) the vanishing point does not coincide with the central vertical axis but does coincide with a visual feature (Figure 1: 3a – 3d); 4) the vanishing point does not coincide with the central vertical axis and does not coincide with a visual feature such as an object, a figure or an architectural detail (Figure 4a – 4d) To avoid possible bias, paintings that can be considered too iconic and insinuating, such as Leonardo’s *Last Supper*, were not selected for the experiment. To compare with Kapoula, Bucci, Yang and Bacci (11), Piero della Francesca’s *Annunciation* was part of the selection.

From literature it is known that human figures and faces (in particular the eyes) tend to attract the beholder’s attention almost automatically (see for instance 3; 6; 9; 25). As most paintings contain human figures, it was important to take into account the extent to which figures affect the attention paid to the vanishing point. Therefore, the selection contained paintings in which figures are absent, few figures can be seen, small figures can be seen, figures can be seen which are semantically and spatially subordinate to the architectural scene, and paintings in which figures are the main semantic content. Although painters used linear perspective as a mode of projection to provide a spatial structure for the content of paintings, a number of paintings used in this experiment by Dutch painter Hans Vredeman de Vries and his followers Hendrik van Steenwijck de Jongere and Dirck van Delen, seem to be made with linear perspective as a means to an end (Figure 1: 1a, 3a, 3b, 3c, 3d, 3f, 4d; Figure 3: 1f, 2e, 3e, 3f). In many of Vredeman de Vries’ paintings and drawings, the vanishing point is more subtly highlighted and coincides with apparently tiny architectural details such as an arch, a window, a portal or a doorway, which are articulated even more strongly by means of colour and by dark and light contrast. Narrative paintings often concern one or more central figures which are also painted relatively large with respect to the whole composition (Figure 1: 1b, 1c, 2a, 2b, 2c.).

### Procedure

All fifteen participants engaged in three tasks during the experiment. In the first task they were shown twenty-four reproductions, six within from each predefined possible position of the vanishing point. In the second task they were shown eight reproductions, two within each predefined position of the vanishing. Paintings were presented to the participants in a randomized order. Each painting was shown for forty-five seconds. Prior to each reproduction, the participant looked at a grey slide with a fixation cross in the upper left of the screen. The participant was asked to fixate on this cross each time. The participants received no other instructions than to just look at the paintings. After each reproduction, they saw a slide on which they were asked to rate each painting successively in terms of whether they liked it or not. This was done using a Likert scale ranging from ‘very much to ‘not at all’. This task was included to make the participant think the experiment was about preferences. As this was not the aim, the data obtained by the Likert scale was not further processed. When looking at the twenty-four reproductions during task I, no instructions were given about the true objective of the experiment or that could suggest the true objective.

After finishing the first task, the participant was shown a slide on which the method of linear perspective was deliberately introduced and the vanishing point explained. On the next slide the participant was instructed to find the vanishing point on eight reproductions of paintings that were shown in a random order, where the four conditions were represented each by two paintings (Figure 3). Participants were asked to locate the vanishing point and to press the space bar as soon as they had found it. This allowed not only to measure the point of fixation at the time when the participant pressed the spacebar, but also the time it took them to identify the vanishing point in the painting.

After completing both tasks, the experimenter asked the participant to perform one final task, which was used to confirm whether the participant had properly understood task number two. In this third task, the participant looked at the eight reproductions from task number two again, but in a different order and in the form of printed black-and-white paper copies. The participant was asked to mark the vanishing point on each of the eight reproductions with an x or by encircling the vanishing point using a pen.

## Results

### Task I

Out of the twenty-four paintings that were used in Task I, the results from sixteen paintings were plotted in the form of heatmaps. The results of eight of the twenty-four paintings were omitted because they contained too much poor-quality data. Three of the eighteen participants’ datasets obtained during Task 1 were not used because of unreliable calibration of the eye pupils. Of the fifteen participants whose data was used, thirteen were female and two were male.

For task I it was expected that the vanishing point would attract more attention when it is at position 1, where the vanishing point is at the central vertical axis and coincides with a visual feature. It was expected that in position 4, in which the vanishing point does not coincide with the central vertical axis or a visual feature, the vanishing point would attract the least attention. It was also expected that in position 2, in which the vanishing point coincides with the central vertical axis but not with a visual feature, the vanishing point would attract more attention than in position 3, in which the vanishing point does not coincide with the central vertical axis, but with a visual feature. However, in position 2, the vanishing point did not attract as much attention as in position 1.

**Figure 2.**
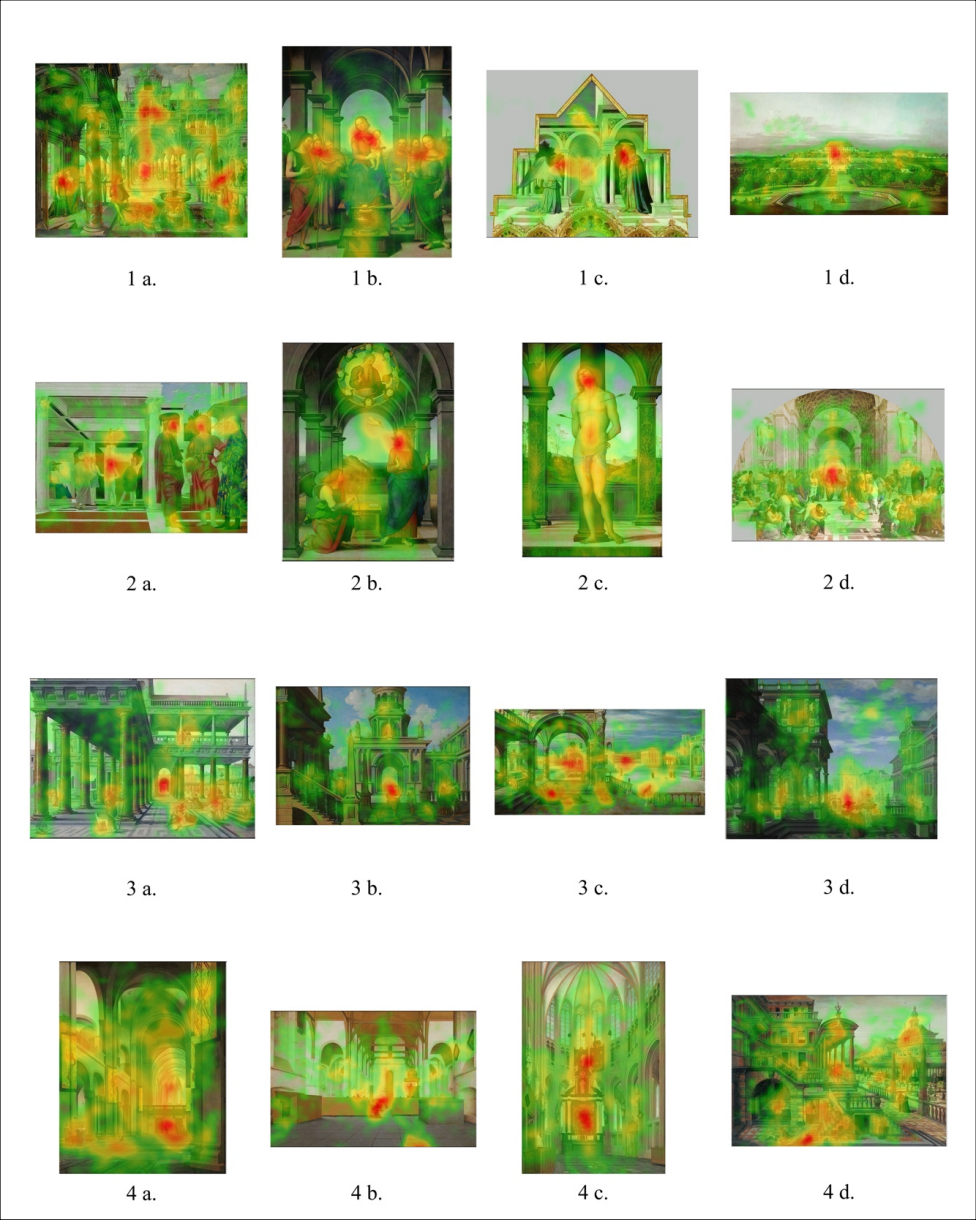
Heatmaps of fixations task I, cumulative results of 15 participants, exposure time: 45 sec per image.

The intensity of fixations on the vanishing point as opposed to other features of the painting was rendered using Eyetrace software and plotted in the form of heatmaps. From these heatmaps it becomes clear that, with respect to the paintings in which the vanishing point is at position 1 (Figure 2: 1a to 1d), participants significantly fixated on the vanishing point area when looking at paintings 1a and 1d as opposed to paintings 1b and 1c. The latter paintings contained notable large human figures and, in line with many eye-tracking studies on paintings containing humans faces and bodies, most of the fixations with regard to these paintings were on those figures and not on the vanishing point area. In the case when the vanishing point is at position 2 (Figure 2: 2a to 2d), the fixations were on the figures and on their faces specifically and hardly at all on the vanishing point area. In the case when the vanishing point is at position 3 (Figure 2: 3a to 3d), fixations were on the vanishing point area but in cases when figures were involved and placed in the vicinity of the vanishing point, as is the case in paintings 3b and 3d, these figures tended to draw the participant’s attention away from the vanishing point area. In the case when the vanishing point is at position 4 (Figure 2: 4a to 4d), there were no significant fixations on the vanishing point area. With regard to this position, participants mainly fixated on other significant details of the paintings, for instance, architectural details such as an altar in a church, or human figures. In the case of the Pieter Saenredam painting 4c, the main area of fixations was the depicted altarpiece, which can be regarded as a painting in a painting (Figure 2: 4c). In line with the definition of this position, these figures and objects did not coincide with the central vertical axis, nor with the vanishing point.

### Task II

Prior to Task II, the participants were asked whether they were familiar with linear perspective. Thirteen stated that they were and two said they were not. With regard to Task II, the data of five of the eighteen participants had to be excluded from the analysis because of poor quality data. Ten of the twelve participants for this selection were female and two were male. The average age of this selection was 23.3 years at the time of the experiment and the mean age was 23.

**Figure 3.**
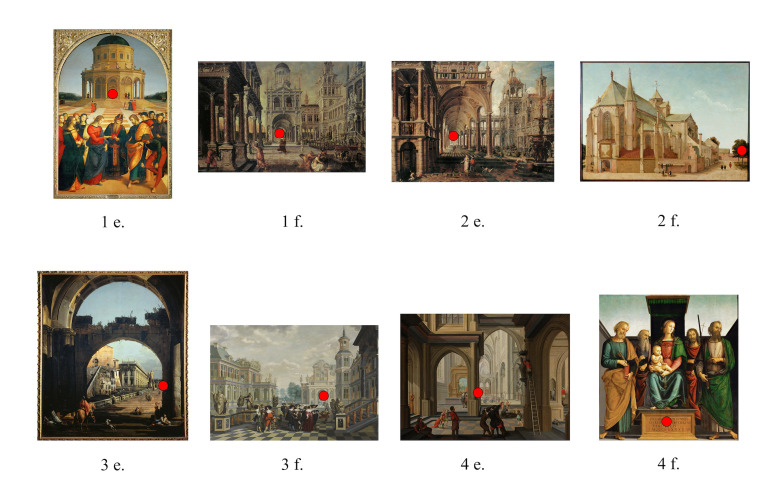
Selection of paintings task II, (vanishing point area indicated by experimenter)

For this task it was expected that the vanishing point would be found within five seconds after exposure when at position 1 and 2, but that it would take longer when at position 3 and significantly longer when at position 4 in which the vanishing point does not coincide with either the central vertical axis nor with a visual feature (Figure 3).

After receiving the instruction to find the vanishing point, participants pressed the spacebar within 5 seconds after exposure in the case the vanishing point was at position 1. However, when the vanishing point is at position 2 it took participants more time to press the spacebar than was expected. In addition, there were also many errors (vanishing point not found). In paintings in which the vanishing point was at position 3, it took slightly longer to press the spacebar than when the vanishing point is at position 1 but less time than when the vanishing point is at position 2. Here, there were no errors. When the vanishing point is at position 4 it took longer to press the spacebar than when the vanishing point is at position 1 and 3 but less time than when the vanishing point is at position 2. However, participants made quite a number of errors (Table 1).

**Table 1.** Results task II & III

Painting	ATS	MTS	VP paper version	
			Yes	No
1e	3.909	2.812	10	2
1f	3.288	3.266	10	2
2e	10.849	9.261	5	7
2f	11.316	8.716	2	10
3e	2.991	2.526	12	0
3f	5.725	5.733	12	0
4e	9.932	8.891	8	4
4f	5.715	6.056	10	2

ATS: Average time in milliseconds until the spacebar is pressed MTS: Mean time in milliseconds until the spacebar is pressed VP paper version: Whether or not the vanishing point was identified on the paper version

### Task III

**Figure 4.**
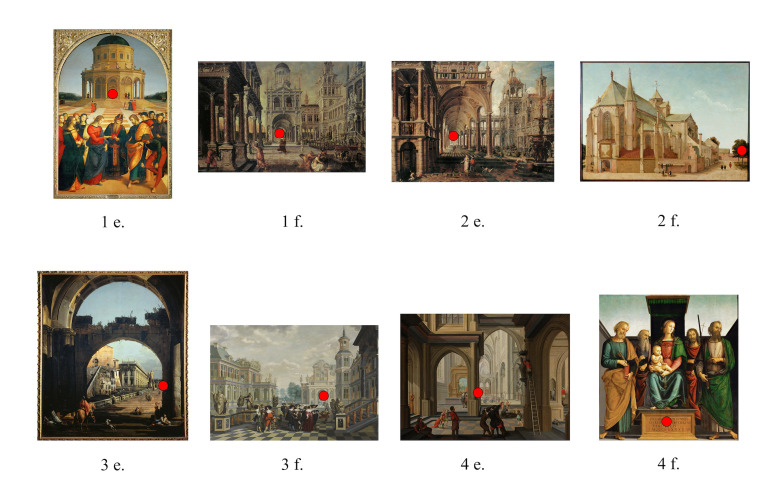
Heatmaps of fixations task II, cumulative results of 12 participants.

This task was to control for whether the participants had correctly understood task II, and was conducted with printed paper versions of the representations. From the data of task II, it was possible to locate the exact point of fixation at the moment the participant hit the spacebar. However, it was not possible to be entirely sure whether this moment indeed coincided exactly with the moment the participant identified the vanishing point. The data shows that for every participant the last fixation on the vanishing point area did not coincide precisely with the exact moment of hitting the spacebar but occurred a few milliseconds before, when the participant may have already moved his or her gaze. The accumulative results plotted as a heatmap still show the extent to which the vanishing point was identified correctly (Figure 4). For instance, it can be seen that the hotspot of fixations for painting 1e is actually slightly away from the vanishing point area. The paper version controlled per participant the extent to which the participant had indeed accurately located the vanishing point.

Furthermore, in task II it appeared that the vanishing point was found relatively quickly, between 3 and 4 seconds, when it coincides with both the central vertical axis and a visual feature. However, when it coincides with the vertical axis only, it not only took relatively long to identify the vanishing point but it was also not identified correctly significantly more often, which became further underscored during task III. The least errors occurred in the case of position 3 in which the vanishing point coincides with a visual feature but not with the central vertical axis, which might indicate that the effect of a visual feature in highlighting the vanishing point is stronger than that of the vertical axis.

Moreover, in task III the identification of the vanishing point appeared to be hardest, both in the experiment as well as in the control test with the paper version, with respect to paintings 2e and 2f. In the case of 2f, however, it is significant that ten of the twelve participants wrongly assumed that the vanishing point must be near the head of the Virgin Mary even though the few orthogonals that can be seen clearly do not point in that direction (see Figure 4, 2f). This could indicate that when the vanishing point cannot be located on the basis of the visible structural-spatial information of the painting, the art historically informed viewer assumes it must coincide with the main content of the painting, in this case the neck of the main figure.

### Exemplary detailed analysis of the results for two paintings:

**Figure 5.**
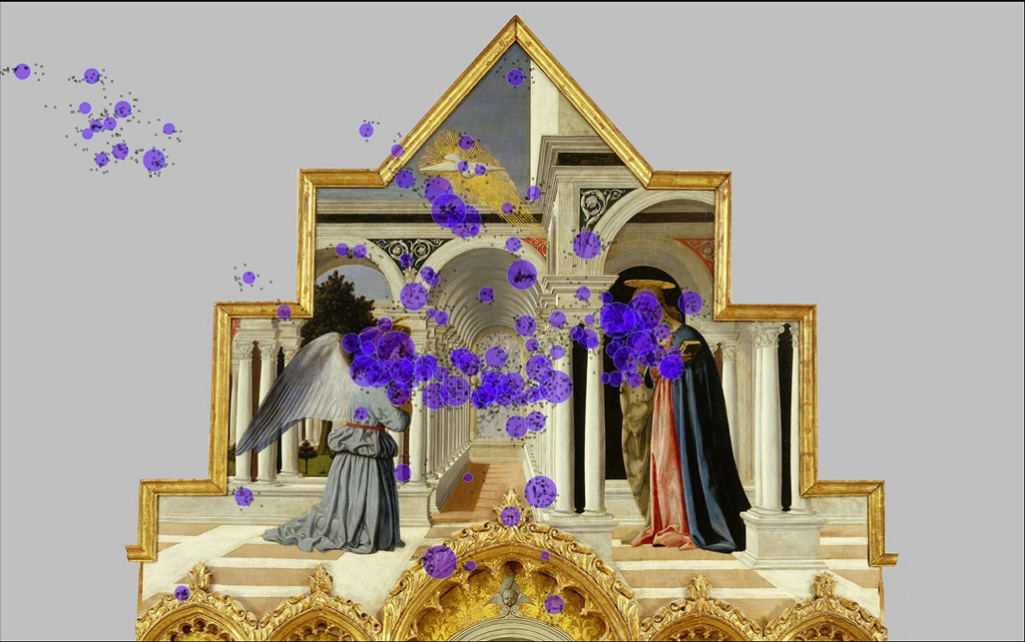
Gaze points and fixations first 2544 ms, cumulative results of 15 participants looking at Piero della Frances-ca, Saint Anthony Polyptych; Annunciation.

**Figure 6.**
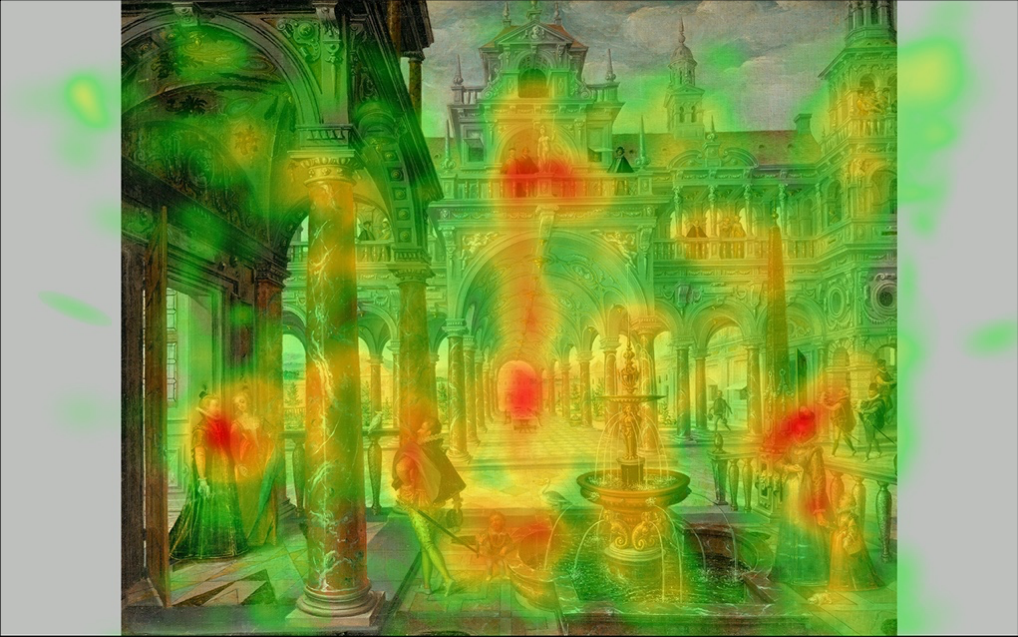
Heatmap of fixations, cumulative results of 15 participants looking at Hans Vredeman de Vries, Palace with distinguished visitors.

On the basis of the general results above, the provisional conclusion can be drawn that the area in which the vanishing point of a perspectival painting resides tends to attract more attention from human beholders when this area coincides with the central vertical axis of a painting. The vanishing point also attracts attention when it coincides with both the central vertical axis, as well as with human figures or objects. However, when human figures do not coincide with the vanishing point, human figures and in particular faces, tend to attract more attention than the vanishing point and other spatial and narrative elements of a painting. To further consider the effect of the vanishing point, the results of two of the twenty-four paintings that could be used in the analysis will now be discussed in more detail. First, the results from Piero della Francesca’s *Annunciation* will be discussed as this allows for comparison with the earlier study by Kapoula, Bucci, Yang and Bacci (11) (Figure 1: 1c, see also figure 5.). Second, the results from Vredeman de Vries’ *Palace with Distinguished Visitors* will be discussed. In this painting in particular one can witness an interesting pattern of viewing that seems to mediate between the vanishing point, which coincides with the central vertical axis and is also emphasized by the architectural element of an arcade, and human figures distributed in the front of the painting and on the balcony (Figure 1: 1a, see also Figure 6). These figures are significantly smaller than for instance those in the Piero della Francesca painting, but they are still definitely notable.

In the pilot study, the vanishing point in this painting was determined as being at position 1. Besides analysing the fixations on the vanishing point in comparison to those on the figures of Mary and Gabriel for the entire beholding time of 45 seconds, the first five fixations of the fifteen participants were specifically considered too. The reason for this is as follows: Kapoula, Bucci, Yang and Bacci (11) looked at the first five fixations of seven participants, of which they eventually calculated the results of only five. In the pilot study in Vienna, the first five fixations of fifteen participants were taken into account. Kapoula, Bucci, Yang and Bacci (11) argue that, of the five participants, four first fixated on the central perspective area, which they defined as the area containing the columns and the farthest plane in between the central arcade of the picture. More precisely, the vanishing point is at the right-hand lower part of what can be specified as the back wall of the centre arcade of the picture. If the central perspectival area is defined as roughly as Kapoula, Bucci, Yang and Bacci (11) did, then from the data collected in the present study it can be stated that the first fixation of 9 out of 15 participants did indeed occur in this area. If, in line with this rough estimation of the central perspectival area, the arch on top of the area is included, even 11 out of 15 first fixations can be regarded as aimed at the central perspectival area. However, in linear perspective the vanishing point is not defined as an area but as an actual determinative point. Based on this definition, none of the first fixations of the participants were on the vanishing point. This was the case in this study as well as in that of Kapoula, Bucci, Yang and Bacci (11). Even though Della Francesca obviously ordered his painting by means of linear perspective, on the basis of the eye tracking results of both above-mentioned studies, it is debatable whether in the first stages of viewing, geometrical perspective affects fixation patterns. The results of both studies rather seem to indicate that the attention is drawn by the central arcade, for instance, because human observers tend to focus on the central parts of images first in the early stages of viewing (17) and because the central arcade is a very obvious visual element framed by two very distinct figures, those of Mary and Gabriel. Many of the second fixations are aimed precisely at Mary (Table 2).

**Table 2.** First five fixations of Piero della Francesca’s Annuncition in relation to content/spatial structure.

Participant	1^st^ fixation	2^nd^ fixation	3^rd^ fixation	4^th^ fixation	5^th^ fixation	End time (ms)
01	Top arch, centre arcade	Mary’s head	Left bottom arch, centre arcade	In depth arch ceiling, centre arcade	Low right corner back wall, centre arcade	1899
02	Right colonnade, centre arcade	Mary’s head	Gabriel’s shoulder	Lintel above centre arcade	Right column, right of centre arcade	1908
03	Halfway middle to the left side of backwall, centre arcade	Mary’s chest	Below Gabriel’s chin	Top golden leaf ornamental border	Wall under left column, left colonnade	1866
04	In depth centre arcade (to the left)	Black space in front of Mary’s face	Left colonnade centre arcade	Column right from Gabriel’s face	Top half back-wall, centre arcade	2199
05	Left column, right of centre arcade	Area below Mary’s hand	Left colonnade, centre arcade	Right colonnade, centre arcade	Ornamental border	1574
06	Left bottom arch, centre arcade	Capital left column ,right of centre arcade	Black area, right arcade	Behind Gabriel’s neck	Grey area left of Holy Spirit	1374
07	In depth arch ceiling, centre arcade	Behind Gabriel’s neck	Right column, left of centre arcade	Mary’s right shoulder	Middle left back wall, centre arcade	1566
08	Top part halo of Holy Spirit	Lintel above columns right of centre arcade	Black area right arcade	Left wing of Holy Spirit	Centre arch, left arcade	1324
09	Centre arch left arcade	Edge right column, right of centre arcade	Mary’s neck	Left colonnade, centre arcade	Right column left of centre arcade	1116
11	Left colonnade, centre arcade	Black area, right arcade	Lower right part back arch, right arcade	Left colonnade, centre arcade	Right colonnade, centre arcade	2183
14	Capital columns right of centre arcade	Black area right of capital columns right of centre arcade	Behind Gabriel’s neck	Black area, right arcade	Mary’s mantle	1849
15	Ornament between left and centre arcade	Capital area, right colonnade	Black area, right arcade	Left lower part back wall centre arcade	Head of Gabriel	1691
16	Left part arch, centre arcade	Black area, right arcade	Capital right column right from centre arcade	Middle point back wall ,centre arcade	Left column left of centre arcade underneath Gabriel’s chin	1566
17	Lower part ornament left of arch above right colonnade	Area between Gabriel’s wing and head	Central ornamental golden border	Gabriel’s shoulder	Extreme left ornamental golden border	2524
18	Gabriel’s halo	Edge of Mary’s mantle	Middle-left back wall, centre arcade	Gabriel’s hands	Lintel below Holy Spirit	2008

Kapoula, Bucci, Yang and Bacci (11) refer to a model proposed by Locher that distinguishes a pre-attentive stage of viewing in which the beholder scans for the global structural aspects of a picture, which can occur in a time even as short as 100ms. For all the fifteen participants in my pilot study, the first five fixations took significantly longer than 100ms. The shortest was 1116ms, while the longest was 2524ms. When all the fixations of any of the fifteen participants between 0 and 2524ms are added, a pattern appears that indicates that the participants’ gaze moves in between the figures of Mary and Gabriel and/or the left and right colonnade of the central arcade. It should be taken into account that the first 2524ms also contain sixth and seventh fixations of participants (Table 2). Taking the total time of viewing into account, which in my pilot study was 45 seconds, the heat-map image of all participants shows that the hotspots of fixations are on or very close to Gabriel and Mary’s heads and to a lesser extent on the Holy Spirit and the back wall of the centre arcade where the vanishing point is located (Figure 2: 1c).

**Figure 7.**
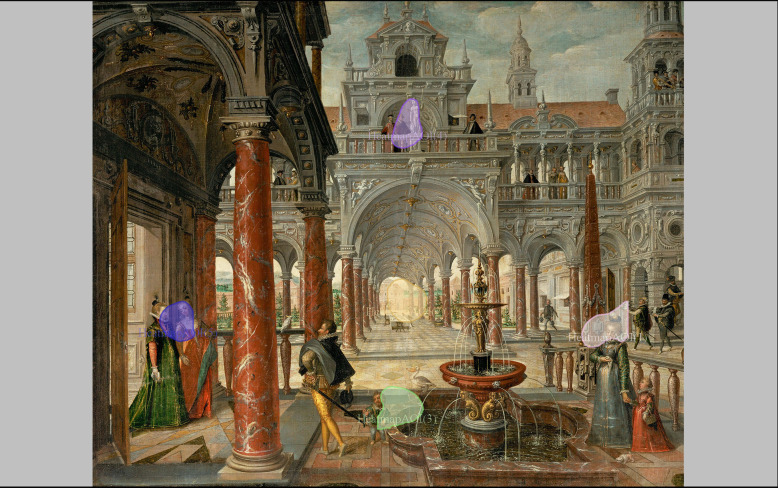
Areas of Interest generated from the highest density of fixations of fifteen participants looking at Hans Vredeman de Vries, Palace with distinguished visitors.

**Figure 8.**
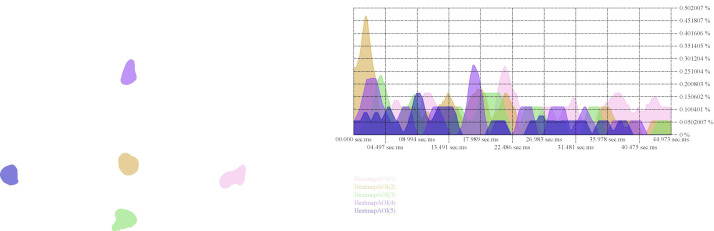
Histogram showing the average amount of fixations (percentage) over time in Areas of Interest.

In many of the paintings and drawings by Hans (and Paul) Vredeman de Vries, the vanishing point appears to be deliberately highlighted by means of making it coincide very specifically with an arch, an arcade or a window, which in the paintings is in turn often highlighted by a dark-light contrast. As compared to Piero della Francesca, the arcade is not a larger area containing the vanishing point but the vanishing point is framed by the much smaller but more brightly lit ending of an arcade which as a visual element frames the vanishing point more precisely (Figure 1: 1a). Vredeman de Vries highlights the vanishing point in nearly all the scenic drawings in the *Perspective* in this way (27). It appears that for Vredeman de Vries, as well as for other Dutch painters of fantasy architecture, linear perspective is a means to an end. It could well have been the case that by means of emphasizing the central vanishing point with architectural and pictorial elements, such as arcades and archways, these painters wanted to draw the beholders’ attention towards the vanishing point to underscore their skills in mastering the principles of central perspective. Fantasy architecture also suits the idea of perspective as a means to an end better than the narrative paintings of Piero della Francesca in which the narrative or the action forms the main content for which linear perspective was a means to geometrically order the space in which this action unfolded. The eye-tracking results from the experiment seem to underscore the above assumption. The heatmap image of the Vredeman de Vries painting (Figure 2: 1a; Figure 6) shows a hotspot of fixations around the central vanishing point. However, there are also hotspots on the two women leaving the room to the left through the doorways, the lady with the child to the right, the figures on the balcony above the arcade and to a lesser extent on the knight and the child to the left of the fountain (see Figure 1: 1a, see for more detail Figure 6). As these figures in terms of pictorial elements such as colour and size merge relatively more into the composition as a whole, as compared to Figure 2: 1c., in which Mary and Gabriel are larger and take up a larger part within the whole composition, the hotspots detected in Figure 2: 1a., allow for a comparison between the attention paid to the vanishing point area and the attention paid to the figures. Therefore, using Eyetrace, Areas of Interest (AOI) were generated from the heatmap of the fixations of the Vredeman de Vries painting using the threshold-based algorithm by Fuhl et al. (8) with a threshold of 60% of the maximum density of the heatmap. Apart from the vanishing point area, it becomes clear that the two figures on the balcony, the two women on the left, the figure to the left of the fountain and the head of the lady to the right of the fountain are Areas of Interest for the participants in this study (Figure 7). The histogram of the five AOIs shows that, of the five AOIs, the one in which the vanishing point resides attracts attention mainly in the first seconds of the viewing process (Figure 8). The comparison between the AOIs further complicates the analysis of the role of the vanishing point area and requires more statistical analysis of the data.

**Figure 9.**
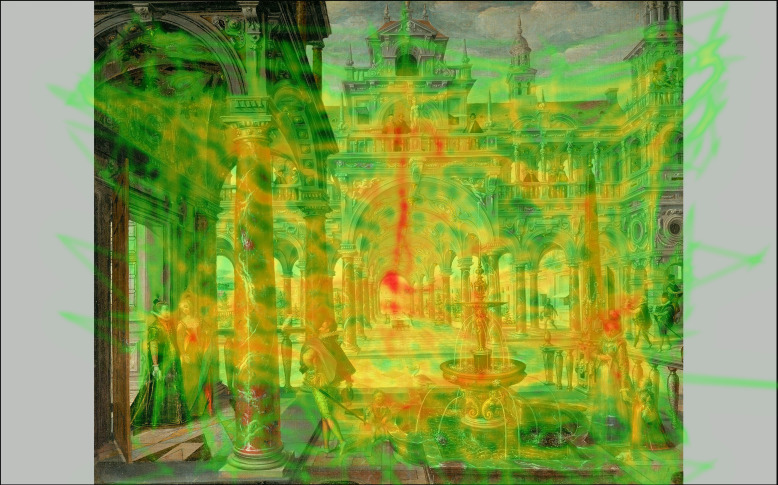
Heatmap of saccades, cumulative results of 15 participants looking at Hans Vredeman de Vries, Palace with distinguished visitors.

Even though this pilot mainly analysed fixations on the vanishing point, I would like to make some remarks about the eye movements between the Areas of Interest in the Vredeman de Vries painting. Figure 9 shows a heatmap of the saccades of the fifteen participants during the 45 seconds they looked at the reproduction of the painting. This is a visualisation showing the average density of saccades among participants in a similar way as heat maps are used to visualise average densities of fixations (15). What is striking is the hot area between the vanishing point area and the figures on the balcony, which partly seems to run in accordance with the central diagonal axis of the ceiling of the arcade. However, the total picture of the saccades does not seem to be a strong indicator that the orthogonals exemplified by the architecture guide the view to the vanishing point. The eye movements between the main content of the painting go from left to right and from top to bottom and sometimes follow a diagonal. Together with the histogram (Figure 8), this image seems to indicate that the viewer starts exploring the painting from its central area in which the vanishing point is also located. In this centre, the arcade, with a strong light-dark contrast also visually forms a strong signifier. From this centre, the viewer appears to scan the image for visually appealing content. The orthogonals exemplified in the architecture, apart from the one in the arcade’s ceiling, do not seem to play a significant role. However, this also needs to be further investigated in future research.

## Discussion

Partly in response to Kapoula, Bucci, Yang and Bacci (11), this pilot study has contributed to our knowledge of the effect of linear perspective on viewing behaviour. The hypothesis underlying this pilot study was that the vanishing point attracts the participant’s view when viewing perspectival paintings and that this effect is stronger when the vanishing point coincides with the central vertical axis of the painting and with a visual feature such as a figure, an object or an architectural detail (like an arch, arcade or a window). It cannot be confirmed whether the vanishing point attracts the participant’s gaze in viewing any kind of perspectival painting. What this pilot study does indicate is that the central vertical axis, as well as visual features, affects the attention paid to the vanishing point. The heatmaps of fixations produced in this study show a clear hotspot of fixations when the vanishing point coincides with a central vertical axis and a visual feature. The results of task II indicate that, when the vanishing point coincides with a visual feature but not with the central vertical axis, the effect of a visual feature might be even stronger than the vertical axis. However, when visual features such as figures appear in paintings as the main content and when they are of considerable size, the attention paid to the vanishing point is less or its location becomes harder to reconstruct. Therefore, this pilot has not been conclusive as to whether the viewer is guided towards the vanishing point (and through that to significant content) by means of the perspectival structure of the painting or whether it is simply the figures and architectural details (a head, a figure, a door, an arcade, a temple etc.) placed on the vanishing point that first attract attention and guide the viewer towards the vanishing point. This is particularly so because the Vredeman de Vries painting (Figure 6) shows that an arcade, for instance, forms a visual detail in the painting with a dark-light contrast, as well as an effect of framing: visual features that likely by themselves attract attention and might therefore obscure the extent to which the viewer is actually attracted by the vanishing point area. With regard to the Piero della Francesco painting (Figure 1, 1c), the area in which the vanishing point resides is also framed, in this case by the colonnade of the arcade which is in turn framed by the figures of Mary and Gabriel. In the first instances of viewing, attention is paid to the architectural feature of the arcade as well as to the figures of Mary and Gabriel but there is no clear indication that viewers are attracted immediately to the vanishing point area (Figure 5). More-over, a possible bias for the centre of the composition also cannot be excluded. In general, though, most of the attention with regard to this painting appears to be paid to the faces of Mary and Gabriel and to a lesser extent to the vanishing point area (Figure 2, 1c).

In the task where participants were deliberately instructed to find the vanishing point (Task II), they could identify this point in nearly all cases except in the case of the Perugino painting (Figure 4, 2f), in which there was relatively little visual information about the orthogonals leading to the vanishing point and where the painting was largely covered by figures. None of the participants were able to locate the vanishing point for this painting correctly; only two participants located it correctly in the control task with the printed versions of the reproduction (Figure 4, 2f; Table 1). This could indicate that in this case when the location of the vanishing point cannot be inferred from architectural details in the painting, viewers familiar with linear perspective probably assume the vanishing point must be near or at the main part (for instance, the head) of the central figure in the painting. The size of figures may also affect the viewer in another way in that the larger the figures are, the easier they will be recognized and draw attention. In that case, their cultural significance will probably also have a greater effect on the viewer, for instance, when the viewer immediately recognizes a figure such as the Virgin Mary, the archangel Gabriel or Christ. Prior knowledge about the way the painting was composed as well as related expectations with regard to the relation between compositional structure and content may further affect the viewer (18).

There are a number of questions that have not been answered fully in this pilot. Firstly, it has not become completely clear at what point during the viewing process the vanishing point attracts the attention of the viewer and whether, or to what extent, the vanishing point is a kind of anchor point to which the viewer regularly returns while looking at the painting. Secondly, it has not become clear to what extent the perspectival structure underlying perspectival paintings, such as the visual pyramid of which all lines converge in the vanishing point and which in paintings is emphasized, for instance, by the architectural details depicted, guides the view, as it were, to the vanishing point. Thirdly, since in this pilot reproductions of paintings were used as stimuli, the effect of the actual size of paintings has not been taken into account. Furthermore, the effect of colour, brightness and luminance has not been analysed. The above makes clear that further research and statistical analysis is necessary to understand how the perspectival structure of the painting in relation to its visual content affects the viewer. Future research could, for instance, focus on the effect of colour, size, luminance and contrast applied to the figures and objects, as well as on knowledge about what these figures and objects mean, in relation to the underlying compositional structure of the painting. Moreover, to understand the extent to which the orthogonals exemplified in the architecture of the painting guide the view, follow-up research is necessary that also considers the saccades of the viewer. As was indicated by the heatmap of the saccades of the Vredeman de Vries painting (Figure 9) as well as the histogram of the AOIs of that painting (Figure 8), when viewing a perspectival painting, viewers apparently do not follow the orthogonals exemplified in the architecture but tend to navigate between the AOIs from left to right and from top to bottom. This could indicate that, even though viewers are aware of the fact that they are looking at an illusion of three-dimensional space, they still navigate the perspectival image as a two-dimensional surface. Further research must also provide more insight into the extent to which the vanishing point area, as emphasized by a visual feature, such as in Vredeman de Vries’ paintings, might function as an almost inescapable visual anchor point for the viewer when navigating the image. This pilot study suggests that generating AOIs from the data would provide a sound method for such further research and statistical analysis. The example of the Vredeman de Vries painting (Figure 6) shows how such AOIs highlight which visual features were significant in the viewer’s viewing process. When analysed more deeply in relation to the viewer’s saccades and the histogram of the viewing process, such data could contribute to our understanding of how the viewing process with regard to perspectival paintings unfolds and what the role is of the visual feature that coincides with the vanishing point in that process. The histogram (Figure 8) indicates that attention paid to the vanishing point area was highest in the early instances of viewing but the extent to which these results might have been biased by, for instance, the fact that this area coincides with the central part of the painting is not clear. Finally, I would like to emphasize the importance of distinguishing between working with digital reproductions and working with actual paintings. This relates to all the above-mentioned points that I addressed for future research. It is very likely that, for instance, the size of the paintings and the museum context will affect the viewer when viewing perspectival paintings. To understand the effect of linear perspective and its structuring elements such as the vanishing point, analyses should therefore ideally be carried out using real paintings as stimuli.

For the time being, it might be concluded that painters like Vredeman de Vries were probably aware of what this pilot study indicates, namely that a theoretical point such as the vanishing point needs to be visually highlighted to make the viewer aware of its existence. The fact that Vredeman de Vries appears to apply this principle consistently could indicate that for him this emphasis was a deliberate means to show and emphasize to his viewers how well and how precisely he had mastered the method of linear perspective.

### Ethics and Conflict of Interest

The author declares that the contents of the article are in agreement with the ethics described in http://biblio.unibe.ch/portale/elibrary/BOP/jemr/ethics.html and that there is no conflict of interest regarding the publication of this paper.

### Acknowledgements

This study was made possible thanks to a fellowship granted to me by Professor Raphael Rosenberg from the Lab for Cognitive Research in Art History at the Department of Art History at the University of Vienna. I am very grateful for this fellowship.

I further wish to thank Dr Johanna Aufreiter, Dr Hanna Brinkmann, Dr Luisa Reitstätter, Dr Eva Specker and Dr Klaus Speidel for instructing me in using the eye-tracking hard- and software. I want to thank Sara Brusnica, Nicolas Kleinschmidt and Felicia Leu for their assistance during the experiment.

## Appendix

### Captions of paintings shown to participants in tasks I, II, III.

**Table:**

1a	Hans Vredeman de Vries, *Palace with distinguished visitors*, 1596, oil painting, 137 x 164 cm., (Vienna, Kunsthistorisches Museum Wien).
1b	Piero della Francesca, *Saint Anthony Polyptych; Annunciation*, 1460-1470, panel, 338 x 230 cm., (Galleria Nazionale d’Umbria).
1c	Perugino*, Fano Altarpiece (Madonna and Child Enthroned with Saints John the Baptist, Peter and Paul, Francis, Louis of Toulouse, Michael Archangel and Mary Magdalene),* 1497, tempera on panel, 262 x 215 cm., (Fano, Chiesa di S. Maria Nuova).
1d	Bernardo Bellotto, *Schlosshof Castle: View from Garden*, 1758-1761, oil on canvas, 136 x 216 cm., (Vienna, Kunsthistorisches Museum Wien).
1e	Raphael, *The Marriage of the Virgin*, 1504, oil on panel, 174 x 121 cm., (Milan, Pinacoteca di Brera).
1f	Hans Vredeman de Vries, Paul Vredeman de Vries & Pieter Isaacsz*, David and Bathseba*, 1602, 123 x 158 cm., (Berlin, Staatliche Museen zu Berlin – Preuβischer Kulturbesitz - Gemäldegalerie).
2a	Piero della Francesca, *The Flagellation*, 1459-1460, tempera on panel, 58.4 x 81.8 cm., (Urbino, Galleria Nazionale delle Marche).
2b	Perugino, *Annunciation*, c. 1498, oil on panel, 212 x 172 cm., (Fano, Chiesa di Santa Maria Nova).
2c	Perugino, *Saint Sebastian*, c. 1490, oil on panel, 176 x 116 cm., (Paris, Musée du Louvre).
2d	Raphael, *School of Athens*, 1509-1510, fresco, 500 x 770 cm., (Vatican City, Stanza della Segnatura).
2e	Dirck van Delen, *Iconoclasts in a church*, 1630, oil on panel, 50 x 67 cm., (Amsterdam, Rijksmuseum).
2f	Perugino, *Madonna and Child with Saints Peter and Paul*, 1493, oil on panel, 186 x 172 cm., (Vienna, Kunsthistorisches Museum).
3a	Hans Vredeman de Vries, *Architectural Caprice with Figures*, 1568, oil on panel, 84.5 x 118.5 cm., (Bilbao, Bilbao Fine Arts Museum).
3b	Dirck van Delen, *Architectural Scene: A Palace Court front*, 1627, oil on panel, 63.5 x 88.1 cm., (Providence, Museum of Art, Rhode Island School of Design).
3c	Hendrick van Steenwijck the Younger, *The Courtyard of a Renaissance Palace*, 1610, oil on copper, 40.2 x 69.8 cm., (London, The National Gallery).
3d	Dirck van Delen, *An Architectural Fantasy*, 1634, oil on panel, 46.7 x 60.5 cm., (London, The National Gallery).
3e	Hans Vredeman de Vries, & Paul Vredeman de Vries, *Palace architecture with lovers and bathers*, c. 1596, oil on canvas, 138 x 186 cm., (Vienna, Kunsthistorisches Museum).
3f	Dirck van Delen, *Elaborate Palace Courtyard with Elegant Company*, c. 1635, oil on panel, 116.5 x 166.7 cm., (Private collection).
4a	Pieter Janszoon Saenredam, *View of the Nave and Choir of St. Mary’s Church in Utrecht, seen from the west*, 1641, oil on panel, 121,5 x 95 cm., (Amsterdam, Rijksmuseum).
4b	Pieter Janszoon Saenredam, *Interior of St. Odulphus Church in Assendelft, seen from the choir to the west*, 1649, oil on panel, 49 x 75 cm., (Amsterdam, Rijksmuseum).
4c	Pieter Janszoon Saenredam, *Cathedral of Saint John at 's-Hertogenbosch*, 1646, oil on panel, 128,9 x 87 cm., (Washington DC, National Gallery of Art).
4d	Hans Vredeman de Vries, Lazarus in front of the palace of the rich man, 1572, oil on panel, 52 x 72,4 cm., (Lemgo, Weserrenaissance Museum Schloss Brake).
4e	Pieter Janszoon Saenredam, *St. Mariakerk Utrecht*, 1659, oil on panel, 44 x 63 cm., (The Hague, Mauritshuis).
4f	Bernardo Bellotto, *Capriccio with the Camidoglio*, 1746, oil on canvas, 132,5 x 117 cm., (Parma, Galleria Nazionale di Parma).
